# Celiac disease and metabolic syndrome: from risk to a preventive opportunity, in the perspective of children’s health

**DOI:** 10.3389/fnut.2026.1751559

**Published:** 2026-01-23

**Authors:** Gianvincenzo Zuccotti, Valeria Calcaterra, Virginia Rossi, Alessandra Vincenti, Federica Loperfido, Paola Baldassarre, Beatrice Maccarini, Alessio Fasano, Hellas Cena

**Affiliations:** 1 Department of Biomedical and Clinical Science, University of Milano, Milano, Italy; 2 Pediatric Department, Buzzi Children’s Hospital, Milano, Italy; 3 Department of Internal Medicine and Therapeutics, University of Pavia, Pavia, Italy; 4 Laboratory of Dietetics and Clinical Nutrition, Department of Public Health, Experimental and Forensic Medicine, University of Pavia, Pavia, Italy; 5 Division of Pediatric Gastroenterology and Nutrition, Department of Pediatrics, Massachusetts General Hospital for Children and Harvard Medical School, Boston, MA, United States; 6 Department of Nutrition, Harvard T.H. Chan School of Public Health, Boston, MA, United States; 7 Clinical Nutrition Unit, ICS Maugeri IRCCS, Pavia, Italy

**Keywords:** cardiometabolic risk, celiac disease, children, gluten-free diet, metabolic syndrome

## Abstract

**Background:**

Celiac disease (CD) is a chronic immune-mediated enteropathy triggered by gluten ingestion in genetically predisposed individuals and is increasingly diagnosed in childhood. Growing evidence suggests an association between CD and metabolic syndrome (MetS), potentially mediated by chronic inflammation, intestinal dysbiosis, oxidative stress, and micronutrient deficiencies. In addition, although a gluten-free diet (GFD) is essential for intestinal recovery, its frequent reliance on ultra-processed, energy-dense products may adversely affect metabolic health, particularly in pediatric patients.

**Objective:**

This narrative review aims to examine the relationship between CD and MetS, with a specific focus on pediatric populations, by analyzing shared pathophysiological mechanisms, the metabolic impact of a GFD, and preventive nutritional strategies to reduce long-term cardiometabolic risk.

**Methods:**

A narrative review was performed using PubMed and Scopus databases, focusing on studies published in the past 15 years. Search terms included “Celiac Disease,” “Metabolic Syndrome,” “Child,” “Adolescent,” “Risk Factors,” and “Prevention.” Among 229 identified papers, 43 were selected after critical appraisal. Evidence was synthesized on epidemiology, mechanisms, dietary effects, and preventive strategies.

**Results:**

Studies indicate that MetS prevalence in CD ranges from 3 to 11% at diagnosis and may rise to 14–29% after 1 year on a GFD, particularly in adults. In children, complete MetS is rare, though isolated components, central adiposity, dyslipidemia, and hypertension, are increasingly observed. Mechanistically, gluten-induced barrier disruption, inflammation, dysbiosis, and nutritional imbalances contribute to systemic metabolic alterations. Adherence to a Mediterranean-style GFD emphasizing whole, naturally gluten-free foods reduces cardiometabolic risk.

**Conclusion:**

CD and MetS share interconnected inflammatory and metabolic pathways. While GFD remains essential for CD management, it necessitates tailored nutritional guidance and metabolic monitoring. Early lifestyle-based interventions—promoting balanced diet quality, micronutrient adequacy, and physical activity, offer key opportunities to prevent metabolic complications in children with CD.

## 1 Introduction

Celiac disease (CD) is an immune-mediated systemic disorder triggered by gluten ingestion in genetically susceptible individuals, characterized by a variable combination of gluten-related symptoms, CD-specific antibodies, HLA-DQ2 or HLA-DQ8 haplotypes, and enteropathy (1). CD represents the most common immune-mediated enteropathy in Western countries, with increasing recognition attributable to improved diagnostic tools and targeted screening of at-risk populations (2–4).

Although the global prevalence is estimated at approximately 1% in the general population, marked geographic variability exists, with higher rates reported in Northern Europe, parts of the Middle East, and North Africa (3–7). Conversely, CD remains likely underdiagnosed in several regions, particularly in Southeast Asia and Sub-Saharan Africa, due to limited epidemiological data and restricted access to diagnostic resources (2–4).

The clinical spectrum of CD has broadened substantially over recent decades. While classical presentations with malabsorption, chronic diarrhea, and failure to thrive still occur, predominantly in younger children, non-classical, oligosymptomatic, and asymptomatic forms now predominate (3, 4). Extra-intestinal manifestations such as iron-deficiency anemia, micronutrient deficiencies, delayed puberty, osteoporosis, neurologic symptoms, dermatologic conditions, and hepatic abnormalities are increasingly recognized (4, 8, 9). CD is also more prevalent among specific high-risk groups, including patients with autoimmune diseases (4, 10–14), chromosomal abnormalities (4, 11, 15), first-degree relatives of affected individuals (4, 16), and those with unexplained anemia or osteoporosis (4, 8, 17). Notably, the median age at diagnosis has increased to approximately 7 years, reflecting both evolving clinical phenotypes and enhanced case finding beyond early childhood (3, 4, 18). Importantly, CD can no longer be considered exclusively a disease of undernutrition, as a substantial proportion of children are now diagnosed while overweight or obese, a pattern increasingly reported in pediatric cohorts (4, 6, 18–23).

In parallel with these changes, metabolic syndrome (MetS) has emerged as a major public health concern in children and adolescents, largely driven by the global rise in pediatric obesity (24, 25). MetS comprises a cluster of cardiometabolic risk factors that substantially increase the likelihood of developing type 2 diabetes (T2DM) and cardiovascular disease (CVD) later in life (24, 26, 27). In the absence of universally accepted pediatric MetS criteria (24, 28), prevalence estimates are largely classification-dependent and therefore variable; on average, MetS affects around 3% of the general pediatric population, increasing markedly among children with overweight/obesity (24, 29, 30). Ethnic disparities have also been documented, with higher rates observed in Hispanic populations and distinct clustering of metabolic risk factors in other ethnic groups (24).

Against this background, growing evidence suggests an emerging overlap between CD and obesity-related comorbidities (20, 23). In particular, although a gluten-free diet (GFD) is essential for disease management, it may be associated with unfavorable changes in body composition and metabolic profile in some patients, potentially contributing to an increased prevalence of overweight and obesity among individuals with CD (20, 23). In addition, chronic intestinal inflammation and altered gut permeability in CD may influence systemic metabolic pathways, including those involved in hepatic and cardiometabolic regulation along the gut–liver axis (10). This convergence is clinically relevant, as CD remains frequently under-recognized despite its high prevalence, especially when presenting with non-classical or silent phenotypes that may coexist with metabolic risk factors (1–4, 18, 20).

This review aims to examine the risk of developing MetS in pediatric patients with CD by synthesizing current clinical and epidemiological evidence, exploring potential pathophysiological mechanisms, evaluating the impact of a GFD, and discussing how these insights can shift CD from a cardiometabolic risk to a preventive opportunity in the perspective of children’s health, emphasizing early nutritional and preventive strategies to support long-term cardiometabolic well-being. A clearer understanding of the intersection between CD and MetS may support improved risk stratification and inform targeted screening and follow-up strategies in children and adolescents with CD (2, 23, 31).

## 2 Methods

### 2.1 Study design

A narrative review of the literature was conducted to explore the association between CD and MetS in pediatric populations, with particular attention to epidemiology, shared pathophysiological mechanisms, the impact of the GFD, and preventive strategies (32, 33). The aim of this review was to summarize and critically interpret available evidence rather than exhaustively identify all published studies. Nonetheless, core methodological elements, such as transparency in the search strategy and study selection, were incorporated to enhance clarity and reproducibility.

### 2.2 Search strategy and eligibility criteria

A comprehensive literature search was conducted in PubMed and Scopus, with English-language publications prioritized for accessibility. When relevant, non-English studies were evaluated based on their abstracts. The search included articles published over the past 15 years. Search terms were used alone and in combination and included: (“Celiac Disease” [MeSH]) AND (“Metabolic Syndrome” [MeSH]) AND (“Child” [MeSH] OR “Adolescent” [MeSH] OR “Pediatrics” [MeSH]) AND (“Risk Factors” [MeSH] OR “Prevention” [MeSH] OR “Primary Prevention” [MeSH]). The search yielded 229 records; after removal of duplicates, 144 titles and abstracts were screened, and 85 full-text articles were retrieved for review. Fifty-nine articles were excluded at screening because they were not pertinent to the scope of the review. Following full-text review, 42 articles were excluded due to insufficient metabolic data, unclear diagnostic criteria for CD or MetS, or a focus exclusively on nutritional aspects without cardiometabolic implications, resulting in 43 studies being included.

Although the primary scope of this review was pediatric, a small number of adult-focused studies were intentionally retained when they offered mechanistic or clinical insights relevant to children or were useful for comparison with pediatric populations (e.g., metabolic effects of the GFD, hepatic or cardiometabolic outcomes, shared autoimmune mechanisms).

**Figure 1:**
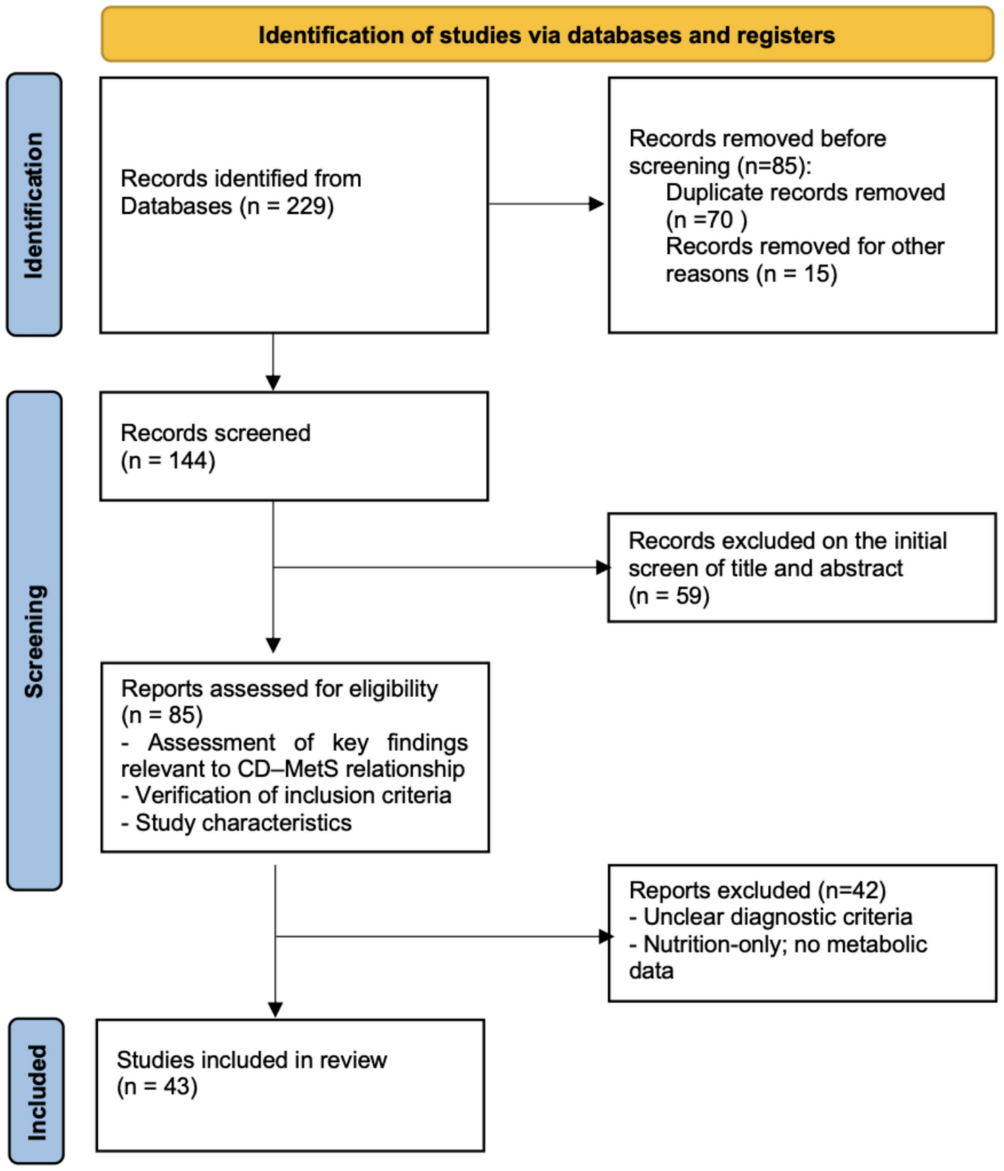
PRISMA-style flowchart summarizing identification, screening, eligibility, and inclusion of studies for this narrative review. CD, celiac disease; MetS, metabolic syndrome.

The study selection process is illustrated in Figure 1, which depicts the identification, screening, eligibility assessment, and final inclusion of studies considered in this review. Additional papers identified through reference lists were incorporated when deemed relevant, in order to strengthen the comprehensiveness and clarity of the overall presentation.

### 2.3 Study appraisal

Eligible studies were qualitatively appraised according to their design. For observational studies, we considered clarity of case definition, adequacy of sample size, appropriateness of metabolic outcome measures, and control of confounding. Systematic reviews and meta-analyses were evaluated for search completeness and transparency, while guidelines and consensus documents were assessed for clarity and relevance to pediatric practice. No studies were excluded based solely on methodological quality, although quality features were taken into account during interpretation.

### 2.4 Data extraction and synthesis

Data extraction was performed independently and focused on study design, population characteristics, diagnostic criteria for CD and MetS, metabolic outcomes, and key findings. Owing to heterogeneity across studies, a qualitative synthesis was undertaken. Themes were defined *a priori* based on review aims (epidemiology, mechanisms linking CD and metabolic dysfunction, metabolic effects of the GFD, and preventive approaches), while additional subthemes (body composition, lipid profiles, insulin sensitivity, liver involvement) emerged during analysis. Findings were synthesized descriptively, emphasizing consistencies, discrepancies, and remaining gaps in evidence.

## 3 Common pathophysiological mechanisms

CD is not only an autoimmune enteropathy but also as a systemic disorder with relevant metabolic consequences. Gluten exposure rapidly increases intestinal permeability within hours to a few days, primarily through gliadin, which activates toll-like receptor 2 and 4 (TLR2 and TLR4) in macrophages, leading to NF-κB overexpression and the release of pro-inflammatory cytokines such as TNF-*α* (34–37). This mechanism has also been documented in pediatric patients with CD (34–37), supporting an early disruption of epithelial barrier function. Gliadin also prolongs epidermal growth factor receptor activation, thereby contributing to proliferative alterations (35). CD is characterized by a persistent pro-inflammatory state that may persist even in the absence of dietary gluten, reflecting intrinsic epithelial fragility and heightened responsiveness of both innate and adaptive immune cells. In genetically predisposed individuals, gluten ingestion further amplifies this process via zonulin-mediated disassembly of epithelial tight junctions. Indeed, gliadin-induced zonulin release and signaling in intestinal epithelial cells causes cytoskeletal rearrangement, phosphorylation of myosin light chain kinase, and subsequent cytoskeletal contraction. These changes disrupt and internalize tight junction proteins, widening paracellular spaces and allowing the passage of larger molecules, including gliadin peptides, across the epithelial barrier. Zonulin, secreted by intraepithelial cells and lamina propria macrophages in response to gliadin, further destabilizes tight junctions, while pro-inflammatory cytokines such as IL-1β, TNF-*α*, IFN-*γ*, and IL-15 exacerbate this dysfunction, creating a vicious cycle of barrier disruption and inflammation (38). The breakdown of tight junction integrity permits luminal antigens, dietary peptides, and microbial products to translocate into the lamina propria, perpetuating mucosal and systemic immune activation. This chronic inflammatory environment not only drives intestinal injury but also promotes systemic metabolic dysregulation, contributing to insulin resistance, dyslipidemia, and other features of MetS (34–37).

The gut microbiota represents another critical component of the pathophysiological cascade in CD. Numerous studies have shown that patients with CD exhibit dysbiosis, characterized by compositional shifts across the oral, duodenal, and fecal microbiota. These include an increased abundance of Proteobacteria, particularly *Escherichia coli* and Neisseria, alongside a reduction in Firmicutes (e.g., Lactobacillus, Streptococcus) and Actinobacteria (e.g., Bifidobacterium) (35). Such changes reflect the depletion of beneficial, anti-inflammatory species and the overrepresentation of pro-inflammatory taxa, including certain strains of Bacteroides and *E. coli* (39–43). While these mechanisms are well characterized in adult and experimental models, their direct contribution to metabolic alterations in pediatric CD remains only partially explored (41–43). These microbial imbalances can exacerbate intestinal barrier dysfunction by disrupting tight junction integrity, degrading the protective mucus layer, and altering mucosal immune responses. In addition, the microbiota produces a wide array of metabolites, such as short-chain fatty acids, bile acid derivatives, and lipopolysaccharides, that directly influence epithelial function, host energy metabolism, and systemic inflammation (39–43), as suggested by emerging pediatric evidence (41–43). Furthermore, alterations in the mucus barrier further compromise intestinal defense. In CD, the duodenal mucus shows reduced hydrophobicity compared with healthy controls, and this abnormality persists despite adherence to a GFD (44). Consequently, the mucus becomes less effective at limiting contact with luminal antigens, while changes in glycocalyx glycosylation favor greater bacterial adherence to the epithelial surface (35). Collectively, these shifts not only aggravate intestinal pathology but also contribute to insulin resistance, dyslipidemia, and other cardiometabolic risks, reinforcing the bidirectional relationship between dysbiosis and systemic inflammation.

Nutritional factors represent a relevant mechanistic link between CD and MetS when considered in the context of intestinal barrier dysfunction, chronic inflammation, and systemic metabolic regulation. In CD, villous atrophy and persistent mucosal inflammation impair epithelial integrity and nutrient absorption, creating a milieu in which dietary composition exerts amplified metabolic effects. Dietary patterns frequently observed in patients with CD, particularly after initiation of a gluten-free diet, are often characterized by a higher intake of fats and simple sugars and a lower intake of fiber, which can further promote intestinal permeability, dysbiosis, and low-grade systemic inflammation, thereby contributing to insulin resistance, dyslipidemia, and endothelial dysfunction (45–47). While these associations are well established in adult populations, pediatric data remain heterogeneous (48). In addition to diet composition, malabsorption-related micronutrient deficiencies constitute a key pathway linking CD to metabolic disturbances. Deficiencies in iron, vitamin D, folate, and B-group vitamins, commonly reported in CD due to villous damage, have been associated with impaired mitochondrial energy metabolism, altered lipid and glucose homeostasis, dysregulation of homocysteine metabolism, and endothelial dysfunction (49, 50). Through these mechanisms, nutritional deficiencies contribute to an adverse cardiometabolic profile independently of caloric intake (49, 50). Taken together, nutritional factors in CD do not represent peripheral or incidental exposures but rather act in concert with intestinal injury and immune activation to amplify shared inflammatory and metabolic pathways underlying MetS. This integrated perspective clarifies the relevance of nutritional elements within the CD–MetS framework and underscores the importance of dietary quality and nutritional monitoring in mitigating long-term cardiometabolic risk in patients with CD (41, 48–50). However, this perspective should be regarded as hypothesis-generating rather than evidence-based in pediatric populations, given the limited availability of long-term longitudinal studies assessing hard metabolic outcomes.

Chronic immune activation in CD also causes oxidative stress (OS). Excessive generation of reactive oxygen species (ROS) overwhelms endogenous antioxidant defenses, namely superoxide dismutase, glutathione peroxidase and glutathione, causing cellular damage to proteins, lipids and DNA (41, 51, 52). In CD, two case–control studies have demonstrated a dysfunction of the glutathione redox cycle and a reduced capacity for lipid hydroperoxide detoxification: Ferretti et al. (50) reported increased levels of lipid peroxidation products and impaired antioxidant defenses in patients with active celiac disease, while Stojiljković et al. (53) documented a significantly altered oxidized/reduced glutathione ratio (GSH/GSSG) in pediatric patients with biopsy-proven CD, both in small intestinal mucosa and peripheral blood, compared with healthy controls (50, 53). ROS not only damage enterocytes, further weakening epithelial integrity and facilitating antigen translocation, but also activate transcription factors that upregulate pro-inflammatory mediators, perpetuating immune activation (41). Beyond its role in sustaining intestinal inflammation, oxidative stress directly contributes to key metabolic syndrome parameters. Excessive ROS generation interferes with insulin signaling by impairing insulin receptor substrate phosphorylation and promoting inflammatory kinase activation, thereby favoring insulin resistance. In parallel, ROS reduce nitric oxide bioavailability, promote vascular inflammation, and increase endothelial activation, leading to endothelial dysfunction and impaired vascular reactivity. While these mechanisms are well established in adult populations, direct evidence in pediatric CD remains limited and is largely extrapolated from adult and experimental studies (41, 48). Through these mechanisms, oxidative stress represents a mechanistic link between chronic immune activation in CD and the development of cardiometabolic alterations characteristic of MetS. An emerging therapeutic perspective involves modulating the oxidative balance through dietary strategies, such as regulating the n-3/n-6 PUFA ratio, although data on CD remain limited (41, 48).

Importantly, the GFD, which is indispensable in the management of CD, has been increasingly implicated in the progression of metabolic disruptions. Improvement in permeability can be seen no less than 4 to 8 weeks, therefore, while mucosal healing improves nutrient absorption, it also allows for greater caloric utilization (35). At the same time, the nutritional profile of many industrial gluten-free products, which are often characterized by a high glycemic index, elevated fat content, and low fiber, contributes to weight gain, visceral adiposity, and dyslipidemia (54). Alterations in lipid metabolism are commonly reported after GFD initiation, with increased triglyceride levels and reduced HDL cholesterol consistently observed, while data on glycemic control and blood pressure remain heterogeneous (48, 55).

**Figure 2:**
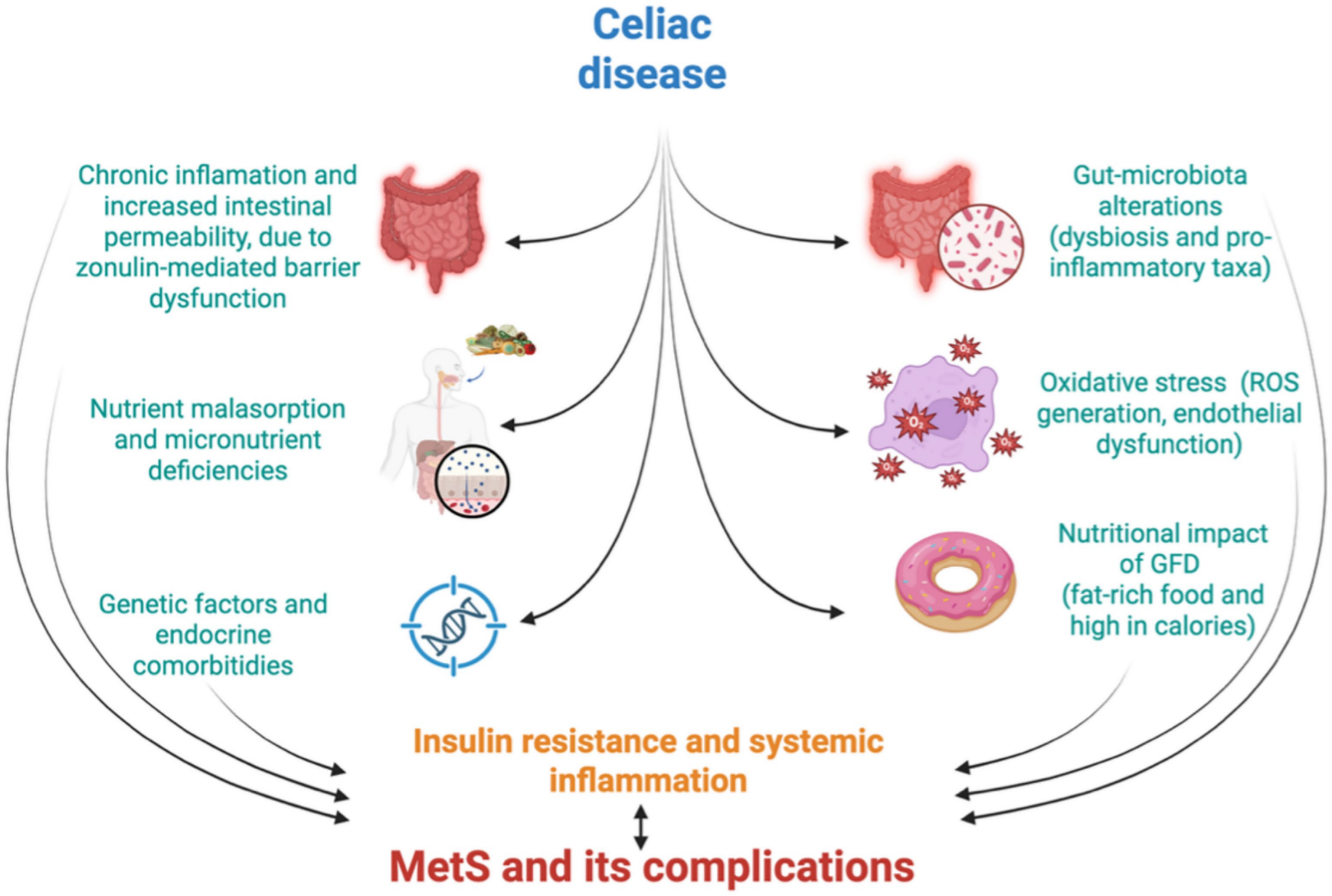
Common pathophysiological mechanisms linking celiac disease (CD) with metabolic syndrome (MetS). The framework integrates intestinal barrier dysfunction, chronic inflammation, gut dysbiosis, oxidative stress, malabsorption-related nutrient deficiencies, gluten-free diet (GFD) nutritional composition, genetic susceptibility, and endocrine comorbidities as interconnected contributors to cardiometabolic risk. Some mechanisms are supported by evidence from pediatric studies, whereas others are extrapolated from adult cohorts or experimental models and should be interpreted as biologically plausible hypotheses. This figure provides an integrative conceptual overview and has not been directly validated in pediatric populations.

Other contributing factors include genetic predisposition and overlapping molecular pathways. Shared HLA haplotypes (such as HLA-DQ2 and HLA-DQ8) and common gene expression signatures between CD and metabolic/autoimmune conditions (e.g., type 1 diabetes (T1DM), autoimmune thyroid disease, inflammatory bowel diseases), observed in both adult and pediatric populations, suggest that genetic background plays a role in susceptibility to both disorders (56). Endocrine comorbidities, particularly autoimmune thyroid disease and T1DM, further magnify metabolic risk in CD populations (57, 58). Moreover, environmental triggers, including infections and dietary exposures beyond gluten, as well as immune-mediated extraintestinal manifestations (i.e., hepatic steatosis and MASLD) are increasingly recognized as contributors to the metabolic burden in CD (58). Figure 2 summarizes the shared mechanisms linking CD with MetS.

## 4 Effects of the gluten-free diet on MetS parameters

Over the past decade, the relationship between CD and MetS has received growing attention. However, prevalence estimates remain highly variable due to differences in study design, diagnostic criteria, patient demographics, duration of GFD exposure, and regional or ethnic backgrounds. These sources of heterogeneity, which complicate interpretation of cardiometabolic outcomes in pediatric celiac disease, are summarized in Table 1.

**Table 1:** Sources of heterogeneity in the assessment of metabolic syndrome and cardiometabolic risk in pediatric celiac disease.

Dimension of heterogeneity	Explanation (including pediatric evidence)
Definition of metabolic syndrome	There is no universally accepted definition of MetS in children. Pediatric studies use modified adult ATP-III criteria, age- and sex-adjusted cut-offs, z-scores, or do not diagnose MetS at all. As a result, reported prevalence in children with CD is inconsistent and not comparable across studies.
Metabolic endpoints assessed	Most pediatric studies evaluate only individual metabolic components (lipids, waist circumference, insulin resistance indices) rather than defining MetS as a composite syndrome. Thus, metabolic burden in children is inferred indirectly.
Insulin resistance assessment	HOMA-IR, fasting insulin and other indices are used inconsistently, often without puberty-adjusted reference thresholds. Reported “insulin resistance” in children may reflect developmental physiology rather than celiac disease or GFD effects.
Lipid abnormalities	Reporting varies greatly: some studies report only HDL-C, others include full lipid panels or lipid ratios. Pediatric data after GFD show possible ↑ triglycerides and ↓ HDL-C, but inconsistent reporting prevents reliable comparisons.
Blood pressure	BP is often omitted. When included, percentile-based pediatric norms are inconsistently applied. Consequently, whether CD or the GFD influences BP in childhood remains unclear.
Hepatic involvement	Pediatric evidence shows potential liver steatosis in CD, but hepatic assessments (ALT, ultrasound) are rarely integrated into MetS evaluation. This likely leads to underestimation of cardiometabolic burden in children.
Timing of metabolic assessment	Follow-up timing varies widely (baseline only, 6–12 months, or several years). Short follow-up may miss delayed metabolic effects of the GFD, especially during growth and puberty.
Duration and nutritional quality of GFD	Adherence and macronutrient composition of the GFD are highly variable in children. Processed-food-based GFD vs. naturally gluten-free Mediterranean patterns may lead to different outcomes, but most studies do not capture this information.
Population characteristics	Pediatric cohorts differ in age, pubertal status, sex, ethnicity, baseline BMI and nutritional status. Lack of stratification by puberty compromises interpretation of metabolic changes.
Regional or ethnic background	Pediatric cohorts come from different geographical and ethnic contexts (European, Middle Eastern, North American), which differ in CD phenotype, baseline nutrition, and cardiometabolic risk → regional variability likely contributes to inconsistent findings but is rarely controlled for in analyses.
Study design	Evidence is predominantly cross-sectional and observational → scarcity of longitudinal pediatric cohorts prevents determining whether CD or GFD *causes* metabolic changes.

ALT, alanine aminotransferase; ATP-III, Adult Treatment Panel III criteria (National Cholesterol Education Program); BMI, body mass index; BP, blood pressure; CD, celiac disease; GFD, gluten-free diet; HDL-C, high-density lipoprotein cholesterol; HOMA-IR, Homeostatic Model Assessment of Insulin Resistance; MASLD, metabolic dysfunction–associated steatotic liver disease; MetS, metabolic syndrome; NAFLD, non-alcoholic fatty liver disease; TG, triglycerides; US, ultrasound; WC, waist circumference.

Overall, MetS is reported in approximately 3–11% of adults at the time of CD diagnosis and increases to 14–29% following 1 year of GFD adherence (55, 59–61). These prevalence estimates derive from selected cohorts and are influenced by heterogeneous diagnostic criteria, age ranges, geographic settings, and both the duration and timing of assessment in relation to gluten-free diet exposure. In pediatric populations, isolated components of MetS are frequently observed, but the full syndrome remains relatively uncommon. Accordingly, prevalence data and metabolic outcomes should not be extrapolated across age groups without explicit qualification. Adults with CD generally present with a lower prevalence of MetS at diagnosis compared with controls (3–5% vs. 12–13%), yet this prevalence rises substantially following GFD initiation (61). By contrast, children and adolescents usually maintain healthier BMI percentiles due to growth-related energy requirements, though subtle increases in visceral fat and changes in body composition still warrant close monitoring (48, 62).

Alterations in body composition, particularly increased visceral adiposity, are strong predictors of cardiometabolic risk at any age and appear more relevant than BMI alone (63, 64). These findings highlight the importance of age-specific nutritional counseling and continuous metabolic surveillance for both pediatric and adult CD patients (23, 65). The metabolic effects of GFD are mediated by multiple factors, including improved intestinal absorption after mucosal healing, lifestyle modifications such as reduced physical activity (48, 64) and increased intake of calorie-dense gluten-free products, which often contain higher levels of fats, saturated fats, simple carbohydrates, and salt to improve palatability and texture, resulting in excessive caloric intake and adverse lipid profiles (65). Additionally, GFD-related shifts in the gut microbiota may alter lipid and glucose metabolism, further influencing metabolic homeostasis (48, 62).

Therefore, while strict adherence to a GFD remains the only effective treatment for CD, ensuring mucosal healing and clinical remission, it may also lead to macronutrient and micronutrient imbalances, including both deficiencies and excesses (66). Increasing evidence indicates a rise in overweight and obesity among CD patients, partially due to the hypercaloric content of many commercially available gluten-free foods (67). Consequently, GFD adherence may negatively impact cardiometabolic risk factors such as body weight, serum lipid concentrations, insulin resistance, MetS development, and even atherosclerosis (23).

Taken together, these findings suggest that GFD adherence can influence several key parameters of MetS. It should be noted that the strength of evidence varies considerably, ranging from observational pediatric studies to adult cohorts and mechanistic or experimental data, which should not be interpreted as equivalent. The variability in study results and the often unspecified composition of GFD contribute to ongoing debate regarding the precise role of GFD in MetS pathogenesis and their components. Moreover, most studies have assessed individual metabolic parameters in isolation rather than the full MetS spectrum, further limiting causal interpretation of GFD-related metabolic changes.

Importantly, interpretation of GFD-related metabolic effects is further limited by the frequent lack of detailed dietary characterization in the available literature. In many studies, the nutritional composition of the gluten-free diet, including degree of food processing, glycemic index, fiber content, fat quality, and overall dietary patterns, is not systematically reported. Consequently, it remains difficult to disentangle the metabolic impact of intestinal healing and improved nutrient absorption following mucosal recovery from that of dietary quality, ultra-processed food consumption, and lifestyle factors such as physical activity, which are only inconsistently assessed.

### 4.1 Gluten-free diet and central adiposity

The initiation of the GFD may be associated with excess weight gain and increased waist circumference if not carefully monitored. For instance, in pediatric populations, Levran et al. (67) found that initiating a GFD in children with CD was associated with changes in dietary patterns that may promote excess weight gain. Using the Family Eating and Activity Habits Questionnaire, the study identified a shift toward more obesogenic dietary behaviors following the start of the GFD (67). Another case–controlled study that compared children with CD on a GFD to healthy children and adolescents found that the GFD group had greater increases in unhealthy weight and BMI after diet initiation (65). Other analyses, however, have noted a growth and weight benefit in this population, as reported in a retrospective study of pediatric American and Italian patients with CD that noted American children had a lesser chance of being overweight or obese when on a GFD (68). Studies in adults with CD indicate that the GFD provides beneficial nutritional support without causing patients to gain excessive weight (69), although the composition of these diets must be closely monitored. This concept of composition regulation is highlighted in a study of adults with CD placed on a hybrid GFD that also incorporated some aspects of the Mediterranean diet (MD) and noted weight stabilization. It is hypothesized based on these reports that gluten itself is not the cause of weight gain, which is instead attributable to the overall composition of foods consumed (especially degree of processing and sugar content) (69).

In pediatric CD, body composition analyses reveal that children with MetS components exhibit substantially higher total and truncal fat percentages and reduced muscle-to-fat ratios, even when BMI z-scores do not indicate overweight, further underscoring the role of fat quality and distribution in determining risk (59, 62). Additionally, Brambilla et al. (70) evaluated BMI changes in 150 children with CD over a median of 4.4 years on GFD, compared to healthy controls, showing that although CD children typically present with lower BMI at diagnosis (reflective of malnutrition or underweight status) GFD introduction leads to BMI normalization and a reduction in the proportion of underweight individuals, with only minimal increases in overweight or obese categories. In contrast, adults with CD demonstrate greater shifts toward overweight or obesity after GFD initiation, associated with increased triglycerides and fasting glucose levels (70, 71).

### 4.2 Gluten-free diet, glycemia and glycemic parameters

Literature data regarding the effects of the GFD on glycemic parameters are controversial. Studies have shown that patients with CD exhibit a close relationship to endocrine autoimmunity, with 10 to 30% of patients with a concomitant diagnosis of T1DM (72). The relationship between CD and T2DM is less defined. A study examining the prevalence of T1DM and T2DM in 1358 CD patients found that the prevalence of T2DM was similar to that of the general population (73). Another investigation addressing the same issue reported that the prevalence of CD among individuals with T2DM was higher than that observed in the general population (74). In a large prospective study involving mostly asymptomatic children with CD, HbA1c levels increased by 0.6% after 1 year of GFD treatment (75). However, other trials in CD patients found no significant changes in HbA1c (76, 77).

### 4.3 Gluten-free diet, HDL-cholesterol and triglycerides

Evidence on the effects of a GFD on HDL-cholesterol (HDL-C) and triglycerides in children with CD remains heterogeneous. Early reports suggested a reduction in HDL-C after GFD initiation (55), whereas more recent pediatric cohorts generally indicate neutral or favorable lipid responses, including increases in HDL-C and reductions in triglycerides (78, 79).

Sex-related differences have also been described, with higher HDL-C concentrations reported in girls compared with boys following GFD initiation (79). Although these mechanisms have not been specifically investigated in CD, they align with known physiological sex-related variations in lipid metabolism during childhood and adolescence, driven by pubertal stage, hormonal milieu, and body composition (80). Supporting these observations, Forchielli et al. (79) evaluated 235 children and adolescents with CD and noted sex-specific differences at baseline, as well as improvements in triglycerides and HDL-C among those with paired measurements before and after dietary change, suggesting benefits when the GFD is nutritionally balanced. Consistently, a 2016 trial in children with CD and T1DM (53), demonstrated significantly higher HDL-C in those on a GFD compared with controls, whereas a retrospective cohort including individuals with CD (57) reported reductions in HDL-C, highlighting that metabolic outcomes may vary depending on age, disease phenotype, and dietary composition.

To date, no meta-analyses have specifically evaluated the effects of a GFD on HDL-C and triglyceride levels in pediatric patients with CD. Although meta-analyses assessing the metabolic or cardiovascular effects of a GFD are available, these syntheses predominantly include adult or mixed-age populations and do not allow pediatric-specific conclusions on lipid outcomes (81). Consequently, evidence in children with CD remains limited to heterogeneous observational studies, precluding a robust quantitative synthesis. Taken together, current evidence suggests that the impact of a GFD on HDL-C and triglycerides in pediatric CD is not uniform but appears to depend on sex, age, baseline metabolic status, and, critically, the nutritional quality of the GFD.

### 4.4 Gluten-free diet and blood pressure

Blood pressure is a MetS component that needs to be evaluated, and it may depend on an unbalanced dietary pattern. Interestingly, in a study performed on children with CD and receiving a GFD or newly diagnosed children, the first group had blood pressure significantly higher in girls than in boys also compared with the group without a GFD (79). Furthermore, in a prospective study conducted to assess the effects of a GFD on CVD risk among newly diagnosed pediatric CD subjects, no significant data were displayed in regard to changes in blood pressure (82). In a cross-sectional multicenter study involving 114 CD children on GFD, Norsa et al. (23) reported the profile of CVD risk factors including hypertension, but also obesity, abdominal obesity, high LDL-C, high triglycerides and insulin resistance. Notably, among the main prevalent CVD risk factors was hypertension (29.4%) (23).

### 4.5 Gluten-free diet and liver disease

Several systematic reviews and meta-analyses have highlighted that the prevalence of non-alcoholic fatty liver disease (NAFLD) increases from approximately 18% in untreated patients to over 28% after starting a GFD, while the prevalence of MetS rises from 4% to over 21% following dietary treatment (55, 59, 60). These estimates are based on studies using the historical NAFLD definition; more recent frameworks classify hepatic steatosis within the broader category of metabolic dysfunction-associated steatotic liver disease (MASLD), to emphasize its close relationship with systemic metabolic impairment. Although most available data derive from adult or mixed-age cohorts, emerging evidence suggests that both children and adults with CD may be at increased risk of MASLD due to mechanisms such as enhanced caloric and nutrient absorption after mucosal healing, high-energy dietary patterns, dyslipidemia, and systemic inflammation (18, 31). In view of these risks, clinical guidance from the European and North American Societies for the Study of Celiac Disease recommends regular monitoring of liver enzymes, lipid profiles, and cardiovascular risk factors in patients with CD adhering to a GFD (83). These recommendations reinforce the need for continued surveillance beyond intestinal recovery and highlight the importance of an integrated management strategy that incorporates systemic metabolic health.

Table 2 provides a structured overview of definitions, metabolic endpoints, and timing of assessment in key studies evaluating cardiometabolic outcomes following a GFD.

**Table 2:** Summary of prevalence and effects of GFD on components of MetS.

Study	Population	Prevalence of MetS at diagnosis	Prevalence after GFD	MetS definition used	Metabolic endpoints assessed	Timing of assessment	Impact of GFD on metabolic risk	Pediatric vs. Adult differences
Yerushalmy-Feler et al. (64)	Pediatric CD	~40% with ≥1 MetS component	Not evaluated	No formal pediatric MetS definition	Body fat %, WC, BP, TG, HDL-C, glucose	Cross-sectional	Fat percentage predicts MetS risk, not BMI	Body composition more predictive than BMI in children
Norsa et al. (23)	Pediatric CD	8.8% overweight, 5.3% obese	11.5% overweight, 8.8% obese	No formal MetS definition	BMI, LDL, TG, BP, HOMA-IR	Diagnosis vs. follow-up on GFD	Increased LDL, triglycerides, blood pressure, insulin resistance after GFD	Metabolic risk increase less pronounced than adults
Więch et al. (65)	Pediatric CD	Not reported	Not reported	No formal MetS definition	BMI, fat mass, FFM, BCM	Cross-sectional on GFD	GFD associated with higher weight, BMI, fat-free mass, muscle mass, and body cell mass; tendency toward higher fat mass	Pediatric only
Anania et al. (22)	Pediatric CD	No formal MetS prevalence	No formal MetS prevalence	None standardized	BMI, BP, lipids, glucose	Baseline + follow-up	Suggestive metabolic changes, not quantified as MetS	Pediatric only
Sansotta et al. (68)	Pediatric CD	Not reported	Not reported	No MetS criteria applied	Anthropometry (BMI, BMI-z, weight categories)	Baseline + ~ 1 year	GFD changes BMI class; no MetS data	Pediatric only
Forchielli et al. (79)	Pediatric CD (cross-sectional *n* = 205; prospective *n* = 30)	Not reported	Not reported	None	TC, TG, HDL, LDL, BP, BMI, glucose, 24-h diet recall, physical activity normal; BMI	Group 1 yearly; Group 2 during 1st year of GFD	In Group 1 → lipid profile differs vs. general population (girls: TC, TG, LDL, HDL ↑). In Group 2 → TG ↓, HDL ↑, glucose ↑. GFD may contribute to lipid differences; does not clearly improve metabolic profile overall.	Pediatric only
Brambilla et al. (70)	Pediatric CD	Not reported	Not reported	None	BMI changes over time	Longitudinal follow-up	GFD associated with increases in BMI and overweight prevalence in some patients	Pediatric only
Zifman et al. (82)	Pediatric CD	Baseline CVD risk factors measured	Follow-up after GFD	None	Lipid profile (TC, TG, HDL), BMI, glucose, BP	At diagnosis and after ~6–12 months of GFD	Some lipid and glucose parameters improved, others unchanged; overall mixed metabolic change	Pediatric only
Salardi et al. (78)	Pediatrics CD + type 1 diabetes	Not formally MetS	After GFD but with type 1 diabetes	None	Lipid profile (HDL, TC), glucose control	Before and after GFD	Lipid profile negatively influenced by CD; GFD intervention showed effects on HDL	Pediatric only
Barone et al. (69)	Pediatric/Adult CD	Lower BMI than controls at diagnosis	9% shift to overweight/obese on GFD	BMI-focused	BMI, weight status	Longitudinal follow-up on GFD	Mean BMI increases on GFD, but risk of obesity remains low in children	BMI increase more marked in adults
Aggarwal et al. (59)	Adult CD	11.4%	18.2% (1 year GFD)	NCEP-ATP III–based criteria	WC, BP, fasting glucose, TG, HDL-C, liver steatosis	At diagnosis and 1 year after GFD	Increased risk of MetS and fatty liver after GFD	Adult only
Tortora et al. (55)	Adult CD	2%	29.5% (1 year GFD)	Modified NCEP-ATP III	WC, BP, fasting glucose, TG, HDL-C	Baseline and 1 year GFD	Significant increases in waist circumference (WC), blood pressure, glycemia, triglycerides (TG) after GFD	Adult only
Ciccone et al. (60)	Adult CD	3.2%	14.6%	NCEP-ATP III	WC, BP, glucose, TG, HDL-C, liver ultrasound	Diagnosis vs. follow-up on GFD	Increased risk of MetS and hepatic steatosis after GFD	Adult only
Kabbani et al. (61)	Adult CD	3.5%	Not reported	NCEP-ATP III	BMI, diabetes, dyslipidemia, hypertension	Cross-sectional; mixed GFD duration	Lower prevalence of MetS than controls; risk increases after GFD	Adult only
Motazedian et al. (71)	Adult CD	5.8%	11.6% (1 year GFD)	IDF criteria	WC, TG, HDL-C, BP, fasting glucose	Baseline and 12 months GFD	Significant increase of WC and TG levels after GFD	Adult only
Valvano et al. (62)	Adult CD	Similar to general population at diagnosis	Increases after GFD	NCEP-ATP III	BMI, WC, lipid profile, liver steatosis	Baseline and follow-up	GFD ↑ NAFLD, weight gain, and lipid alterations; recommend monitoring liver, weight, and metabolic profile	Adult only

ATP-III, Adult Treatment Panel III criteria (National Cholesterol Education Program); BCM, body cell mass; BMI, body mass index; BP, blood pressure; CD, celiac disease; FFM, fat-free mass; GFD, gluten-free diet; HDL-C, high-density lipoprotein cholesterol; HOMA-IR, Homeostatic Model Assessment of Insulin Resistance; IDF, International Diabetes Federation; MetS, metabolic syndrome; NAFLD, non-alcoholic fatty liver disease; TG, triglycerides; WC, waist circumference.

## 5 Therapeutic and nutritional implications

GFD comprises both naturally gluten-free foods and processed products formulated to contain less than 20 parts per million (ppm) of gluten, in accordance with European regulations (84). Therefore, the exclusion of gluten, a protein complex responsible for the viscoelastic and structural properties of food, poses technological and nutritional challenges (85). To improve texture, mouthfeel, and palatability, manufacturers often incorporate hydrocolloids, fats, and proteins into gluten-free formulations (86). As a result, many commercial gluten-free products show an unbalanced nutritional profile, with higher amounts of saturated fats, sugars, and sodium, and lower levels of protein and fiber compared to their gluten-containing counterparts. These differences can promote excess caloric intake, adiposity, and dysmetabolic alterations, thereby increasing the risk of developing MetS (86).

In recent years, the nutritional trajectory of children with CD has shifted from undernutrition at diagnosis toward more complex patterns of body composition change. While some patients still present with growth impairment (87), others experience normalization or even excessive increases in BMI and fat mass after GFD initiation (88). This variability is strongly influenced by dietary quality: consuming hypercaloric, low-fiber, ultra-processed gluten-free products is increasingly common, often displacing naturally gluten-free foods rich in complex carbohydrates and micronutrients (70). Such patterns not only affect weight status but also contribute to early metabolic alterations, including dyslipidemia, insulin resistance, and visceral adiposity, all components or precursors of MetS (64). Therefore, nutritional counseling and preventive strategies should begin at diagnosis, with special attention to dietary composition and long-term cardiometabolic monitoring in children.

Chronic low-grade inflammation is a noteworthy characteristic of CD, indicating that numerous pediatric patients may experience a phase of untreated inflammation before diagnosis (89). This sustained inflammatory state has been associated with muscle deterioration and sarcopenia (90). In a cohort study involving 111 children diagnosed with CD, 44 of whom also met the criteria for MetS, results showed a significantly elevated BMI z-score (*p* < 0.001), increased sarcopenic indices (*p* = 0.05), a greater percentage of body fat, and reduced muscle-to-fat ratio z-scores (*p* = 0.018). Additionally, the study reported higher systolic blood pressure percentiles (*p* = 0.001), elevated triglyceride levels (*p* = 0.009), and increased triglyceride to HDL-cholesterol ratios (*p* < 0.001) (64). Consequently, children with CD may be at an increased risk for sarcopenic obesity and, therefore, for early-onset cardiometabolic disorders. The diet of children with CD should be a high-quality GFD based on the Mediterranean dietary pattern, emphasizing the consumption of naturally gluten-free foods and pseudocereals such as buckwheat, quinoa, amaranth, and millet, in addition to legumes, fruits, and vegetables (91). This dietary approach is beneficial for enhancing fiber and micronutrient intake, mitigating postprandial glycemic responses, and reducing cardiometabolic risk. The inclusion of certified uncontaminated oats is advisable, as they can safely increase dietary fiber and *β*-glucan intake (92).

Since the MD is strongly associated with positive health outcomes and a reduced incidence of non-communicable diseases, including MetS in pediatric populations, adopting this dietary pattern may help reduce the risk of metabolic complications early in life. Furthermore, promoting adherence to the MD in children with CD could provide a foundation for healthy eating habits and metabolic health across the lifespan (91).

In addition to dietary composition, education for patients and caregivers is fundamental to improve long-term dietary quality. Families should be guided to critically read food labels, assess the nutritional quality of commercial gluten-free products, and understand the implications of cross-contamination (93). In pediatric care, targeted nutritional strategies should include individualized energy prescriptions based on growth velocity and developmental stage, systematic substitution of refined gluten-free staples with higher-fiber and higher-protein alternatives, and comprehensive micronutrient monitoring with supplementation as needed (94). In this context, registered dietitians play a central role by promoting the preparation of meals with naturally gluten-free ingredients, encouraging a reduction in the consumption of ultra-processed gluten-free foods, and supporting the development of sustainable, health-oriented dietary practices.

Micronutrient deficiencies are frequent at diagnosis of CD and may persist during long-term adherence to GFD, even with good compliance. These deficits result from both villous atrophy and nutritional limitations of unfortified gluten-free foods, as staple cereal-based products such as bread, pasta, and flour typically contribute significantly to the intake of iron, folate, calcium, and B vitamins in the general population (95). Gluten-free flours are often unfortified, further increasing the risk of nutrient deficiencies in children with CD (95, 96).

Common deficiencies include iron, vitamin D, calcium, zinc, and fat-soluble vitamins, with vitamin D deficiency being the most prevalent and strongly associated with reduced bone mineral density, impaired growth, and increased cardiometabolic risk (66). Several studies show improvements in bone mineral content and metabolism markers after starting a GFD, but full normalization often requires appropriate supplementation (97, 98). A meta-analysis involving 624 pediatric CD patients and 532 controls confirmed significantly lower 25(OH)D levels and higher rates of vitamin D deficiency compared to healthy peers (99). Intervention studies further suggest that vitamin D and calcium supplementation alongside GFD adherence can normalize biochemical parameters, enhance biochemical and bone health outcomes, although careful monitoring is required to avoid hypervitaminosis D (100).

Current evidence on water-soluble vitamin status in children and adolescents with CD suggests that, overall, deficiencies in B1, riboflavin (B2), pyridoxine (B6), folate, and vitamin C are relatively uncommon and generally comparable between treated and untreated patients, whereas vitamin B12 shows a clear improvement following adherence to a GFD (97). Despite this, dietary intake assessments reveal that several B vitamins are often consumed at levels below dietary recommendations, both in the short and long term (101). In contrast, vitamin C intake is usually adequate or improves with a GFD, and vitamin B12 intake is generally sufficient, with serum concentrations typically within normal ranges (101).

Regarding minerals, children adhering to a GFD often exhibit improvements in calcium, iron, and ferritin compared to untreated patients, although magnesium, selenium, and zinc levels may remain suboptimal (97).

Children with anemia due to iron, folate, or vitamin B12 deficiency may require supplementation alongside a GFD, especially during critical periods of brain development (94). For example, a retrospective cohort study involving 304 newly diagnosed children with CD and iron deficiency showed that ferritin levels increased significantly over 12 months on a GFD, both in supplemented and non-supplemented children, suggesting that iron status can normalize with dietary management alone in many cases (102). However, other evidence highlights that micronutrient deficiencies persist even in children diagnosed early through screening (96). A case–control study of 84 screen-detected children with CD and 443 healthy controls found significantly lower serum levels of hemoglobin, iron, ferritin, vitamin D, zinc, copper, and selenium in the celiac group, with more severe intestinal lesions associated with reduced ferritin and vitamin A (96).

In the context of MetS, several micronutrients play a key role in regulating lipid and glucose metabolism (103). Vitamin D deficiency, in particular, has been associated with increased BMI, waist circumference (WC), visceral adiposity, insulin resistance, and dyslipidemia (104, 105). Moreover, a study conducted on a sample of 6,311 children and adolescents supports these findings, demonstrating that vitamin D deficiency (<30 nmol/L) is significantly associated with an increased risk of obesity, as assessed by BMI, WC, and waist-to-height ratio (WHtR) (106). Vitamin D deficiency is also linked to a reduced HDL cholesterol levels and elevated insulin resistance (106).

Besides, altered iron homeostasis, reflected in elevated serum iron, has been correlated with hyperglycemia and hypertension, whereas biomarkers of iron accumulation are associated with reduced HDL cholesterol (106). Collectively, these findings indicate that micronutrient imbalance could be both a consequence and a driver of early metabolic dysregulation in CD (106).

Effective management of CD involves not only optimizing diet and supplementation but also incorporating physical activity (PA) and lifestyle modifications to reduce the heightened risk of MetS and fatty liver disease, especially after starting a GFD. Observational studies in children with CD suggest that increased engagement in vigorous PA is associated with enhanced lean muscle mass and improved bone health, regardless of the duration of adherence to a GFD. These findings support the inclusion of exercise as a supplementary intervention aimed at promoting musculoskeletal and metabolic health. Furthermore, global surveillance data (*n* = 1.6 million participants) indicates that 81% of youth fail to meet the PA guidelines established by the World Health Organization (WHO) (107). This is particularly alarming during the critical developmental phases of puberty and early adulthood, as the achievement of peak bone mass during these periods is a significant factor in predicting future osteoporosis risk, especially in females. Accordingly, there is a compelling rationale for the systematic incorporation of structured aerobic and muscle-strengthening exercises, along with behavioral counseling and coordinated efforts involving families and educational institutions, into the care settings for individuals with CD.

Therefore, a comprehensive management of children with CD should integrate dietary quality optimization, targeted micronutrient monitoring and supplementation. Regular evaluation of dietary intake, serum nutrient concentrations, bone health, and cardiometabolic parameters is strongly recommended. A personalized, quality-oriented GFD, combined with lifestyle interventions and evidence-based supplementation, is essential to correct nutritional deficiencies, prevent metabolic complications, and support optimal growth, development, and long-term health outcomes.

## 6 Limitations

This narrative review has several limitations that should be acknowledged. First, although the review is intended to focus on pediatric celiac disease, part of the mechanistic and metabolic evidence is derived from adult cohorts or experimental models and has therefore been interpreted as biologically plausible rather than definitively established in children. Second, the assessment of metabolic syndrome in pediatric populations is inherently heterogeneous, as no universally accepted diagnostic criteria exist and studies variably apply modified definitions, z-scores, or evaluate individual metabolic components rather than the full syndrome. Third, interpretation of metabolic outcomes related to the gluten-free diet is limited by the frequent lack of detailed dietary characterization in the available literature. In many studies, the nutritional composition of the GFD, including degree of food processing, glycemic index, fiber content, fat quality, and overall dietary patterns, is not systematically reported, making it difficult to disentangle the metabolic effects of intestinal healing and improved nutrient absorption from those related to dietary quality and lifestyle factors such as physical activity. Fourth, reported prevalence estimates and metabolic outcomes are influenced by differences in study design, population characteristics, duration and nutritional quality of the gluten-free diet, and timing of follow-up, limiting direct comparability across studies. In addition, important pediatric-specific confounders, including age at diagnosis, duration of untreated disease, pubertal stage, growth velocity, baseline nutritional status, and family dietary environment, are inconsistently reported and may substantially influence metabolic trajectories. Finally, most available pediatric data are cross-sectional, with limited longitudinal evidence to support causal or temporal relationships between celiac disease and cardiometabolic risk. Together, these limitations highlight the need for standardized definitions, detailed dietary assessment, and prospective pediatric studies, while warranting a cautious interpretation of epidemiological associations and metabolic outcomes reported in pediatric celiac disease.

## 7 Future perspectives and research

Future research on the relationship between CD and MetS should aim to elucidate the multifactorial mechanisms that link intestinal, metabolic, genetic, and environmental determinants. New risk biomarkers are needed to improve early risk stratification, including inflammatory mediators, oxidative stress markers, lipidomic and metabolomic profiles, and indicators of gut barrier integrity. Identifying such biomarkers may enable more precise monitoring of metabolic alterations in pediatric and adult CD patients, particularly during long-term adherence to a GFD.

The integration of precision medicine approaches offers a promising avenue for future studies. Advances in genetics, microbiota analysis, and nutrigenomics could provide insights into inter-individual variability in metabolic response to the GFD, the influence of specific genetic polymorphisms (e.g., HLA and non-HLA loci), and the impact of microbial composition on energy metabolism and systemic inflammation. Personalized dietary interventions, guided by molecular and microbiome profiling, may optimize GFD composition to improve metabolic outcomes and reduce cardiometabolic complications.

Finally, longitudinal studies are essential to establish causality and to evaluate the long-term effects of the GFD on metabolic health across the lifespan. Large, prospective, multicenter cohorts should integrate nutritional, metabolic, and microbiological data to develop evidence-based, integrated guidelines for the management of CD patients at risk for MetS. These efforts will help define standardized monitoring protocols, promote balanced dietary patterns such as the Mediterranean-based GFD and ensure early preventive strategies in children to mitigate future cardiometabolic risk.

## 8 Conclusion

Clinically, these findings reinforce a dual perspective: while the GFD remains indispensable for intestinal recovery, symptom control, and long-term prevention of celiac-related complications, there is a clear need for early, proactive strategies to minimize iatrogenic metabolic risk. Nutritional counseling at diagnosis should prioritize naturally gluten-free whole foods and discourage excessive consumption of calorie-dense, processed substitutes. Regular monitoring of anthropometric and metabolic parameters, such as weight, waist circumference, blood pressure, fasting glucose/HbA1c, lipid profile, and body composition, should be adapted to patient age and risk level, alongside encouragement of habitual PA. Personalized nutritional management throughout follow-up is essential to balance the risks of undernutrition and excessive weight gain, particularly in pediatric populations.

From a clinical nutrition standpoint, the management of CD in childhood requires an integrated and dynamic approach that extends beyond the mere exclusion of gluten. Periodic evaluation of micronutrient status, including vitamin D, iron, folate, zinc, and selenium, is recommended to ensure optimal growth and prevent deficiencies.

Personalized nutritional counseling, ideally delivered by a multidisciplinary team, should encourage adherence to a Mediterranean-style GFD, emphasizing naturally gluten-free foods rich in fiber and micronutrients while limiting the consumption of ultra-processed products.

Looking ahead, the integration of precision nutrition approaches, accounting for the gut microbiome, HLA genotype, and individual variability in metabolic response to the GFD, may enable increasingly tailored interventions, enhancing therapeutic efficacy and reducing the risk of MetS and cardiometabolic complications in children and adolescents with CD.

Routine assessment of body composition should be integrated into the clinical management of young patients aiming to an optimal celiac care. Due to inconsistent findings across studies, further investigation is necessary to elucidate the effects of a GFD on MetS components in CD; the metabolic profile in these patients results from complex interactions between dietary habits, genetic factors, physical activity, and lifestyle, underscoring the need for comprehensive, multifactorial assessment in clinical management.
